# Individual Colorimetric Observer Model

**DOI:** 10.1371/journal.pone.0145671

**Published:** 2016-02-10

**Authors:** Yuta Asano, Mark D. Fairchild, Laurent Blondé

**Affiliations:** 1 Munsell Color Science Laboratory, Rochester Institute of Technology, Rochester, New York, United States of America; 2 Motorola Mobility, Chicago, Illinois, United States of America; 3 Technicolor, Rennes, France; Monash University, AUSTRALIA

## Abstract

This study proposes a vision model for individual colorimetric observers. The proposed model can be beneficial in many color-critical applications such as color grading and soft proofing to assess ranges of color matches instead of a single average match. We extended the CIE 2006 physiological observer by adding eight additional physiological parameters to model individual color-normal observers. These eight parameters control lens pigment density, macular pigment density, optical densities of L-, M-, and S-cone photopigments, and λ_*max*_ shifts of L-, M-, and S-cone photopigments. By identifying the variability of each physiological parameter, the model can simulate color matching functions among color-normal populations using Monte Carlo simulation. The variabilities of the eight parameters were identified through two steps. In the first step, extensive reviews of past studies were performed for each of the eight physiological parameters. In the second step, the obtained variabilities were scaled to fit a color matching dataset. The model was validated using three different datasets: traditional color matching, applied color matching, and Rayleigh matches.

## Introduction

Human color vision differs from person to person. Not only in terms of color deficiency, but also the large variability that exists among color-normal populations [1]. Human color vision is characterized by a set of three spectral response functions, also known as color matching functions (CMFs). Thus, the variability in human color vision essentially equates to the variability in CMFs. From a physiological perspective, CMFs are composed of lens pigment (and other ocular media), macular pigment, and three types photopigments (L-cone, M-cone, and S-cone). Each component has inter-observer variability, contributing to the overall variability in CMFs.

A vision model for individual observers enables us to predict color matches and match ranges among color-normal populations for a given condition. Assessing the ranges of matches rather than a single, average match is beneficial in many color-critical applications. Nevertheless, most of the proposed observer functions are average observer functions. Examples include CIE 1931 Standard Colorimetric Observer, CIE 1964 Supplementary Standard Colorimetric Observer, and CIE 2006 Physiological Observer (CIEPO06) [2]. In 1989, CIE TC 1-07 proposed the CIE Standard Deviate Observer with the aim to evaluate the range of color mismatches for metameric color pairs due to the change in observer [3–6]. However, several researchers reported the CIE Standard Deviate Observer significantly underestimates real human observer variability [7–9].

The proposed model predicts CMFs of a color-normal population through Monte Carlo simulation. The concept was initially proposed by Fairchild [10] and then Fairchild and Heckaman [11, 12] elaborated the vision model taking four physiological parameters (lens pigment density, peak optical density of macular pigment, λ_*max*_ shifts of L-cone and M-cone) as input. In this study, we propose an observer model with eight physiological parameters to more accurately model individual CMFs in addition to the two parameters inherited from CIEPO06. Identifying variations of each physiological parameter, CMFs of a given population could be generated through Monte Carlo simulation.

## Procedure

The proposed individual colorimetric observer model is expressed as Eq (1).

$$\begin{array}{c} lms-CMFs=f(a,v,d_{lens},d_{macula},d_{L},d_{M},d_{S},s_{L},s_{M},s_{S}) \end{array}$$ (1)

where *a* is an age of an observer (as in CIEPO06), *v* is a visual angle [degree] (field size, as in CIEPO06), *d*_*lens*_ is a deviation [%] from an average for lens pigment density, *d*_*macula*_ is a deviation [%] from an average for peak optical density of macular pigment, *d*_*L*_, *d*_*M*_, and *d*_*S*_ are deviations [%] from averages for peak optical densities of L-, M-, and S-cone photopigments, respectively, *s*_*L*_, *s*_*M*_, and *s*_*S*_ are deviations [nm] from averages for λ_*max*_ shifts of L-, M-, and S-cone photopigments, respectively.

The model has ten input parameters. The first two parameters (age and field size) are the same as CIEPO06 input. They determine the average CMFs for a given age and field size. The eight additional parameters determine the deviation from average for each physiological parameter. These parameters modify the basis functions in CIEPO06 to model individual observers. This parameterization is convenient to keep the average observer model intact. When the eight additional parameters are set to zero, the model becomes exactly same as CIEPO06. No attempt was made to modify CIEPO06 functions and improve the average observer model since it was out of scope of this study. The model output is lms-CMFs (also known as cone fundamentals), the same output as in CIEPO06.

The computational procedure is equivalent to CIEPO06 except for additions of individual variability. More detailed explanations are available in the CIE publication [2]. Note that a Matlab code for CIEPO06 is available at Munsell Color Science Laboratory’s website [13]. For a given observer, there are ten input parameters to the model: *a* is an age of the observer, *v* is a visual angle (field size) [degree] determined by an experimental condition, *d*_*lens*_ is a deviation [%] from an average for lens pigment density, *d*_*macula*_ is a deviation [%] from an average for peak optical density of macular pigment, *d*_*L*_, *d*_*M*_, and *d*_*S*_ are deviations [%] from averages for peak optical densities of L-, M-, and S-cone photopigments, respectively, *s*_*L*_, *s*_*M*_, and *s*_*S*_ are deviations [nm] from averages for λ_*max*_ shifts of L-, M-, and S-cone photopigments, respectively. In the following steps, spectral transmission of lens pigment (and other ocular media), spectral transmission of macular pigment, and spectral sensitivity curves of photopigments are computed individually, and combined in the end to obtain a set of cone fundamentals.

The average spectral optical density of the lens and other ocular media, *D*_*ocul*,*ave*_(λ), is obtained by Eq (2) for an observer between the ages of 20 and 60, and Eq (3) for an observer over the ages of 60.

$$\begin{array}{c} D_{ocul,ave}\left(\lambda \right)=D_{ocul,1}\left(\lambda \right)\left(1+0.02\left(a-32\right)\right)+D_{ocul,2}\left(\lambda \right) \end{array}$$ (2)

$$\begin{array}{c} D_{ocul,ave}\left(\lambda \right)=D_{ocul,1}\left(\lambda \right)\left(1.56+0.0667\left(a-60\right)\right)+D_{ocul,2}\left(\lambda \right) \end{array}$$ (3)

where *D*_*ocul*,1_ represents portion affected by aging, and *D*_*ocul*,2_ represents portion independent from aging. *D*_*ocul*,1_ and *D*_*ocul*,2_ are obtained from Table 6.10 in [2]. Eqs (2) and (3) were derived by Pokorny, Smith and Lutze [14] and adopted in CIEPO06. Note that, in this study, age ranges were extended for Eq (2) to incorporate ages younger than 20. It should be noted that some researchers suggested that the lens pigment density would increase exponentially with age [15, 16]. However, the function adopted in CIEPO06 was kept untouched at this time. The individual spectral optical density of the lens and other ocular media, *D*_*ocul*_(λ), is obtained by Eq (4).

$$\begin{array}{c} D_{ocul}\left(\lambda \right)=D_{ocul,ave}\left(\lambda \right)\left(1+\frac{d_{lens}}{100}\right) \end{array}$$ (4)

The spectral optical density of the macular pigment, *D*_*macula*_(λ), is obtained by Eqs (5) and (6).

$$\begin{array}{c} D_{macula}\left(\lambda \right)=D_{max,macula}D_{relative,macula}\left(\lambda \right) \end{array}$$ (5)

$$\begin{array}{c} D_{max,macula}=0.485e^{-v/6.132}\left(1+\frac{d_{macula}}{100}\right) \end{array}$$ (6)

where *D*_*max*,*macula*_ is the peak optical density of the macular pigment, and *D*_*relative*,*macula*_(λ) is the relative spectral optical density of the macular pigment. The data for *D*_*relative*,*macula*_(λ) are tabulated in Table 6.4 in [2]. Eq (6) without the inter-observer variation component was derived by Moreland and Alexander [17] and adopted by CIEPO06.

The cone absorptance spectra, *α*_*j*_(λ) (j = L, M, or S) for L-, M-, and S-cone photopigments are computed by Eqs (7), (8) and (9). Absorptance is defined as the ratio of the absorbed radiant or luminous flux to the incident flux in the given conditions [2].

$$\begin{array}{c} \alpha_{j}\left(\lambda \right)=1-10^{-D_{max,photopig,j}A_{shift,j}\left(\lambda \right)} \end{array}$$ (7)

$$\begin{array}{c} A_{shift,j}\left(\lambda \right)=A_{j}\left(\lambda -s_{j}\right) \end{array}$$ (8)

$$\begin{array}{ccc} D_{max,photopig,L} & = & \left(0.38+0.54e^{-v/1.333}\right)\left(1+\frac{d_{L}}{100}\right)\\ D_{max,photopig,M} & = & \left(0.38+0.54e^{-v/1.333}\right)\left(1+\frac{d_{M}}{100}\right)\\ D_{max,photopig,S} & = & \left(0.30+0.45e^{-v/1.333}\right)\left(1+\frac{d_{S}}{100}\right) \end{array}$$ (9)

where *D*_*max*,*photopig*,*j*_ is the peak optical density of a given cone type, *A*_*shift*,*j*_(λ) is the shifted low optical density spectral absorbance of a given cone type, and *A*_*j*_(λ) is the average low optical density spectral absorbance of a given cone type. The data for *A*_*j*_(λ) are tabulated in Table 6.6 in [2]. Eq (9) without the inter-observer variation component was derived by Pokorny and Smith [18] and adopted by CIEPO06.

Cone fundamentals in terms of quanta are obtained by combining the three components for each cone type as shown in Eq (10).

$$\begin{array}{ccc} l_{q}\left(\lambda \right) & = & \alpha_{l}\left(\lambda \right)10^{-D_{macula}\left(\lambda \right)-D_{ocul}\left(\lambda \right)}\\ m_{q}\left(\lambda \right) & = & \alpha_{m}\left(\lambda \right)10^{-D_{macula}\left(\lambda \right)-D_{ocul}\left(\lambda \right)}\\ s_{q}\left(\lambda \right) & = & \alpha_{s}\left(\lambda \right)10^{-D_{macula}\left(\lambda \right)-D_{ocul}\left(\lambda \right)} \end{array}$$ (10)

Cone fundamentals in terms of energy (subscript omitted) are obtained multiplying quanta-based cone fundamentals by λ as shown in Eq (11).

$$\begin{array}{ccc} l(\lambda ) & = & \lambda l_{q}\left(\lambda \right)\\ m(\lambda ) & = & \lambda m_{q}\left(\lambda \right)\\ s(\lambda ) & = & \lambda s_{q}\left(\lambda \right) \end{array}$$ (11)

As a final step, the three functions are normalized such that the maximum value of each function is unity. All the computations are done with a wavelength step size of 5 nm in accordance with the data tabulated in [2]. When λ_*max*_ shift is applied in Eq (8), spline interpolation can be used to retrieve the average low optical density spectral absorbance at a wavelength location finer than 5nm interval.

To summarize, the variability in the lens pigment is taken into account in Eq (4), the variability in the peak optical density of the macular pigment is taken into account in Eq (6), the variabilities in the peak optical densities of the photopigments are taken into account in Eq (9), and λ_*max*_ shifts of photopigments are taken into account in Eq (8). This procedure can compute the cone fundamentals for any individual observer with ten input parameters. Matlab code for the proposed model as well as an interactive demo are available at Munsell Color Science Laboratory’s website [19].

## Derivation of Physiological Parameter Deviations

Probability distributions are required for eight physiological parameters to perform Monte Carlo simulation. It was assumed that each physiological parameter formed a normal distribution around the mean. In addition to the model simplification, this would be a practical approach as many studies reported standard deviations. The assumption would be reasonable since a population study showed normal distributions [20]. The standard deviations (SDs) of eight physiological parameters were determined at two steps. Note that Webster and MacLeod worked on identifying individual differences of physiological parameters by performing a factor analysis on Stiles and Burch’s individual observer data [21]. The approach in this study was different from their study in that we derived variations from numerous past studies for each physiological parameter.

### Step 1

Numerous studies that reported inter-observer variability in physiological factors were collected and summarized for each physiological parameter in Tables 1, 2, 3 and 4. Studies were selected based on three criteria: (1) a relatively large number of subjects, (2) widespread and well-investigated measurement methods, and (3) subjects free from visual disorders. Any data involving subjects with potentially impaired vision (e.g., color deficiencies, diabetes, cataracts, etc.) were excluded from the analyses. In general, physiological measurements are preferred to psychophysical measurements since the former is usually more precise than the latter. For psychophysical measurements, the number of repeated measurements for a given subject must be large enough to produce reliable variability estimates. Most studies reported standard deviations (SDs) in an absolute unit, which are inconsistent across studies. The inconsistencies are due to different methodologies, different stimulus sizes (eccentricities), and different stimulus wavelengths used in experiments. Thus, SDs [%] were calculated dividing SDs [an absolute unit] by the corresponding average. For λ_*max*_ shift of photopigments, SDs [nm] were obtained instead of SDs [%]. An SD for each physiological parameter was computed by converting SDs of collected studies to variances, taking an average of the variances, then taking the square-root of the average. The results are shown in Table 5. All the studies were treated equally without weighting upon averaging. This was because methodologies were so diverse that many factors affect accuracy (e.g., experimental setting, number of subjects, number of repetitions, instrument quality, etc), and determining a weight to each dataset would be arbitrary.

**Table 1:** Past studies for variability in lens density. Methods include In Vitro, LOM (lens opacity meter), Purkinje Image, SP (Scheimpflug photography), VECP (visually evoked cortically potential amplitude), SBM (scotopic brightness matching), Sct.Thr. (scotopic threshold), and V’(λ) Analysis.

Authors	Year	Meas. Type	Method	Repetitions	Subjects	Age Range	SD [%]
Mellerio [28]	1971	Physiological	In Vitro	N/A	20 eyes	19 - 66	22.7
De Natale et al. [29]	1988	Physiological	LOM	5	266	7 - 86	17.8
De Natale, Flammer [30]	1992	Physiological	LOM	5	799	12 - 89	18.7
Johnson et al. [31]	1993	Physiological	Purkinje Image	16	40	24 - 77	7.7
Savage et al. [32]	2001	Physiological	Purkinje Image	16	41	18 - 59	24.3
Cook et al. [33]	1994	Physiological	SP	N/A	100	18 - 70	24.5
Werner [34]	1982	Physiological	VECP	N/A	50	0 - 70	22.4
Savage et al. [32]	2001	Psychophysical	SBM	≥3	41	18 - 59	18.6
Lutze, Bresnick [35]	1991	Psychophysical	Sct.Thr.	3	50	20 - 69	19.1
Polo et al. [36]	1996	Psychophysical	Sct.Thr.	3	62	20 - 71	24.3
Wild et al. [37]	1998	Psychophysical	Sct.Thr.	12	51	24 - 83	13.1
Hammond et al. [38]	1999	Psychophysical	Sct.Thr.	2	125	20 - 63	12.6
van Norren, Vos [39]	1974	Psychophysical	V’(λ) Analysis	N/A	50	17 - 30	12.8

**Table 2:** Past studies for variability in optical density of macular pigment. Methods include AF (fundus autofluorescence) and FR (fundus reflectometry).

Authors	Year	Meas. Type	Method	Repetitions	Subjects	Age Range	SD [%]
Delori et al. [40]	2001	Physiological	AF	N/A	159	15 - 80	33.3
Wüstemeyer et al. [41]	2003	Physiological	AF	N/A	109	18 - 75	32.6
Liew etal. [20]	2005	Physiological	AF	N/A	300	18 - 50	39.3
Trieschmann et al. [42]	2006	Physiological	AF	N/A	120	20 - 86	38.0
Delori et al. [40]	2001	Physiological	FR	N/A	159	15 - 80	30.4
Berendschot et al. [43]	2002	Physiological	FR	1–2	289	63 - 73	45.5
Broekmans et al. [44]	2002	Physiological	FR	N/A	376	18 - 75	45.5
Wüstemeyer et al. [41]	2003	Physiological	FR	N/A	109	18 - 75	38.7
Berendschot, van Norren [45]	2004	Physiological	FR	N/A	138	18 - 76	27.1

**Table 3:** Past studies for variability in optical density of L-, M-, and S-cone photopigments. Methods include FR (fundus reflectometry), Rayleigh Match, and CMFs Trans. (transformation from CMFs).

							SD [%]		
Authors	Year	Meas. Type	Method	Repetitions	Subjects	Age Range	L	M	S
Berendschot et al. [46]	1996	Physiological	FR	N/A	10	33.5 ± 9.6	18.3	18.3	
Burns, Elsner [47]	1985	Psychophysical	Rayleigh Match	3	11	23 - 47	14.9	14.9	
Elsner et al. [48]	1988	Psychophysical	Rayleigh Match	10	52	13 - 69	20.0	20.0	
Stockman et al. [49]	1999	Psychophysical	CMFs Trans.	N/A	5	N/A			14.7

**Table 4:** Past studies for variability in λ_*max*_ shift of L-, M-, and S-cone photopigments. Methods include In Vitro (MSP, microspectrophotometry), Rayleigh Match, and Test Sensitivity.

							SD [nm]		
Authors	Year	Meas. Type	Method	Repetitions	Subjects	Age Range	L	M	S
Dartnall et al. [50]	1983	Physiological	In Vitro (MSP)	N/A	7 eyes	34 - 70	5.2	3.5	3.6
Merbs, Nathans [27]	1992	Physiological	In Vitro (MSP)	4	6 - 7 cones	N/A			1.4
Burns, Elsner [51]	1993	Psychophysical	Rayleigh Match	≥2	6	28 - 41	2.1	2.5	
Stockman et al. [49]	1999	Psychophysical	Test Sensitivity	20	5	N/A			1.8

**Table 5:** Standard deviations obtained at step 1 and 2. Scalars are those optimized at step 2. Units of SDs are percentages [%] except for λ_*max*_ shifts [nm]. Density denotes optical density, and Shift denotes λ_*max*_ shift.

	Lens	Macula	Density L	Density M	Density S	Shift L	Shift M	Shift S
Step 1	19.1	37.2	17.9	17.9	14.7	4.0	3.0	2.5
Scalars	0.98		0.50					
Step 2	18.7	36.5	9.0	9.0	7.4	2.0	1.5	1.3

For lens density, it is necessary to define an SD for a given age since age ranges significantly impact SDs. For this reason, the following steps were required. For each study, lens densities and ages of subjects were collected. Then, (1) an age center was set, (2) lens densities of subjects whose ages were ± five years from the age center were extracted, and (3) An SD for a given age center was taken from these lens densities. These steps were repeated until all the available age centers were selected. An SD for a given study was obtained taking an average of all the SDs at available age centers.

For macular pigment, there are abundant studies. More detailed and comprehensive reviews are available from other authors [22–25].

For optical densities of L-, M-, and S-cone photopigments, all three studies in Table 3 reported the same variations in optical densities of L-cone and M-cone photopigments (or did not differentiate the two variations) because it is extremely difficult to separately measure optical densities of L- and M-cone photopigments. Renner et al. obtained optical densities of L- and M-cone photopigments separately by a flicker threshold method involving dichromats [26]. SDs obtained from their data were 17.3% and 23.1% for optical densities of L- and M-cone photopigments, respectively. The data from Renner et al. were not used in this study since they came from subjects with impaired color vision. While the variations in optical densities of L- and M-cone photopigments might possibly differ, it was determined to assign the same variability to L- and M-cone photopigments due to scarce data. Given that the average peak optical densities of L- and M-cone photopigments are the same in CIEPO06, it would be reasonable to treat variations in optical densities of L- and M-cone photopigments in a similar manner.

For λ_*max*_ shifts of L-, M-, and S-cone photopigments, the available data were extremely scarce as shown in Table 4. The study by Merbs and Nathans used genetically reconstituted pigments to measure responses [27]. There are many studies reporting variations of λ_*max*_ shifts related to genetic sequences. Although very insightful, they were not used to derive model parameters. The reasoning is explained in Complementary Insights section.

### Step 2

SDs obtained at step 1 were scaled to fit the variability of a target color matching dataset. The step 2 was necessary because otherwise, the model tended to overestimate variations according to the preliminary simulation. The possible explanation would be that the obtained SDs at step 1 contained not only inter-observer variability but also other uncertainties such as intra-observer variability and instrumental errors. When all the SDs were combined to create a synthetic model, such uncertainties were almost certainly accumulated, which would lead to an overestimation of overall variations.

Scaling each SD independently was computationally very expensive. Therefore, instead, two scalars were optimized: One scalar to scale the variability in lens and macular pigment densities and the other scalar to scale photopigment-related variability. Lens and macular pigments are prereceptoral (pre-retinal) filters and thus easier to measure than photopigments. Besides, the number of studies and the number of subjects were much more for prereceptoral filters than photopigments. Thus, it was expected that the level of uncertainties would be different between prereceptoral filters and photopigments.

**Fig 1:**
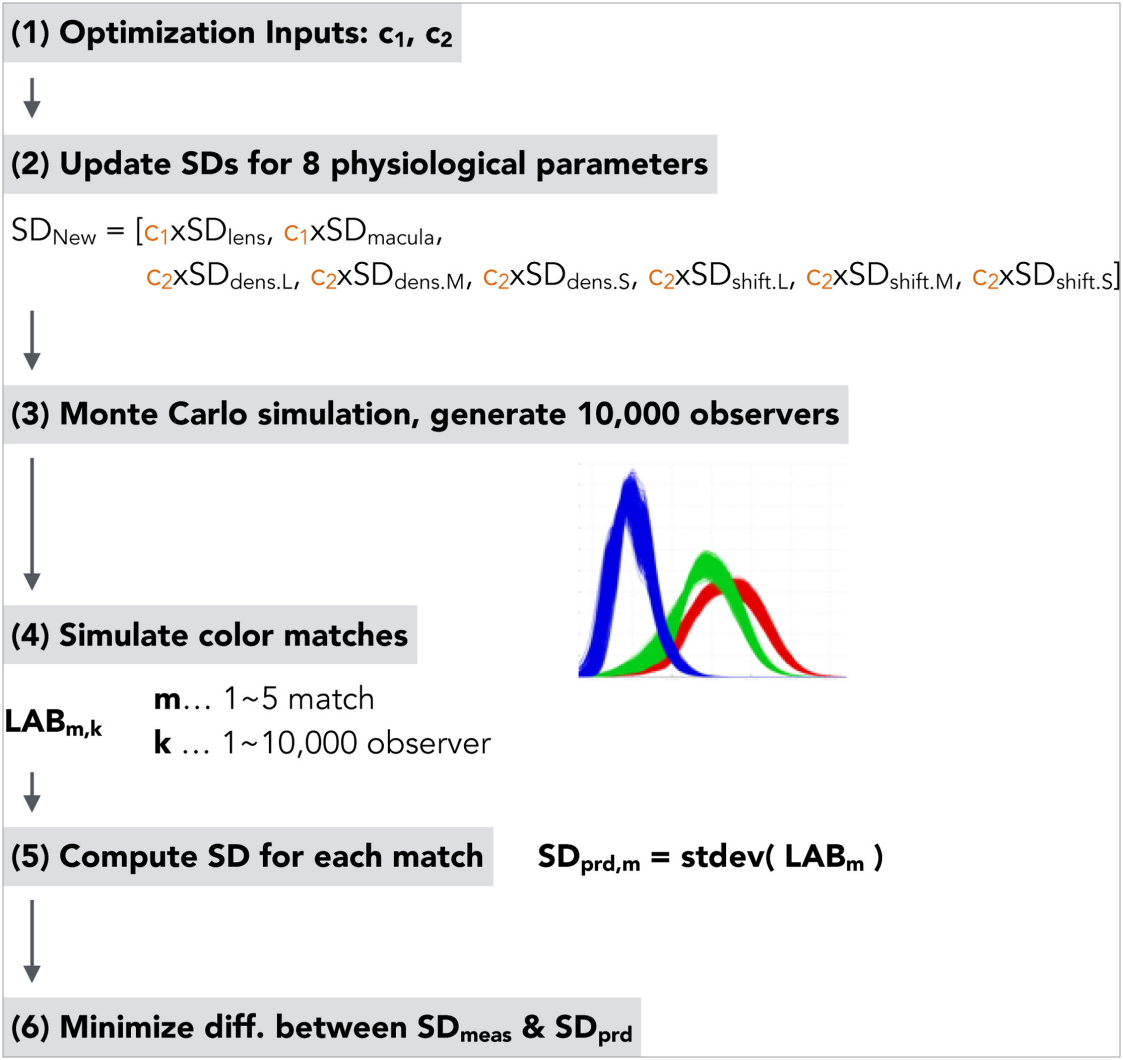
Workflow to optimize two scalars of eight physiological parameters.

The target color matching data resulted from Asano’s Ph.D. work: five color matches with three repetitions for 75 color-normal observers out of 151 observers in total [52]. The rest of the 76 observers’ data were used in the validation part. The nonlinear optimization steps were illustrated in Fig 1 and the explanation follows below.

1. Inputs of the optimization workflow were two scalars varying between 0 and 1.
2. The two scalars updated a standard deviation for each of the eight physiological parameters. The first scalar controlled SDs for lens density and optical density of macular pigment. The second scalar controlled SDs for the rest of parameters (optical densities and λ_*max*_ shifts of photopigments).
3. Using the updated SDs for eight physiological parameters, Monte Carlo simulation was performed and 10,000 sets of CMFs (observer functions) were generated. The age distribution was taken from a list of 76 observer ages. The field size was set to 6.5° (instead of the experimental condition, 8.5°, to better match the average and only investigate individual variations of matches).
4. A color match was simulated for each of the five color matches and for each of the 10,000 observer functions. The predicted (simulated) matches were expressed as CIELAB values to align with experimental results. The detailed color matching simulation procedure is described in Color Matching Simulation Procedure.
5. A standard deviation was computed for each of the five color matches. Only CIELAB a* and b* values were used.
6. Differences between SDs from experimental results (*SD*_*meas*_) and SDs from the simulation (*SD*_*prd*_) were minimized. The objective function is expressed as Eq (12).
  $$\begin{array}{c} \underset{c_{1},c_{2}}{\text{arg}\;\text{min}}\;(\sum_{i=1}^{5}|SD_{meas,i}-SD_{prd,i}\left(c_{1},c_{2}\right)\left|\right) \end{array}$$ (12)
  where *c*_1_ and *c*_2_ are the two scalars, *i* is an index representing one of the five color matches. The distance metric in Eq (12) is known as city block distance (also referred to as Manhattan distance). It was chosen simply because what was minimized was clear, and the city block distance mostly yields results similar to Euclidean distance. The results at step 2 are shown in Table 5 together with results from step 1. In fact, the optimized scalars using Euclidean distance were [0.994, 0.491] as opposed to [0.98, 0.50] for city block distance. The SDs obtained at step 2 were those adopted by the proposed model. The variability estimates in prereceptoral filters were very close to those obtained at step 1 while variability estimates in photopigments were scaled down as much as 50% possibly due to the fact that reported results would contain more uncertainties.

### Complementary Insights

In addition to the original purpose, there are complementary insights from past studies summarized in Table 6.

**Table 6:** Possible factors that might affect physiological parameters. Plus marks indicate factors that are incorporated into CIEPO06. Open circles indicate factors that potentially exist but have not been quantified yet.

			Photopigment	
	Lens	Macula	Density	Shift
Age	+		∘	
Field Size		+	+	
Diabetes	∘			
Smoking	∘			
Dietary Intake		∘		
Gender		∘		
Race/Ethnicity		∘		
Body Fat Percentage		∘		
Genetics			∘	∘
Pupil Entry			∘	
Retinal Illuminance			∘	

Age is the most significant factor contributing to lens optical density (as in CIEPO06). However, age alone cannot explain all the individual variability. Age explains 47% [53] (or 50% [54]) of lens variations. Large variability in lens density exists even for different people of the same age. Two other possible contributors would be smoking and diabetes. Smoking increases lens optical density due to its oxidant effects on the lens and also increases the vulnerability of the lens [38]. The increase is dose-dependent, and it persists even after stopping smoking [38]. Diabetic patients exhibit accelerated yellowing of lens due to elevated plasma glucose levels that may accelerate glycosylation of lens proteins [35].

The change in field size (visual angle) produces systematic shifts in the peak optical density of macular pigment (as in CIEPO06). In addition, dietary carotenoids intake, gender, ethnicity, and body fat percentage are reported to change macular pigment density. The macular pigment consists of two types of carotenoids: lutein and zeaxanthin. As human cannot produce lutein and zeaxanthin, increasing lutein and zeaxanthin dietary intake would increase macular pigment density [55, 56]. Macular pigment density is higher in men than women, following the explanation that the ability to transport lutein and zeaxanthin from blood into eye would be greater in men than in women [44, 57–60]. Macular pigment density was higher in Asians than Caucasian observers [60], and lower in Blacks than Caucasian observers [61]. Macular pigment density decreases as the percentage of body fat increases since adipose tissue and retina compete for uptake of lutein and zeaxanthin [62]. Any other factors that modify the efficiency to transport the consumed lutein and zeaxanthin to the retina could affect the macular pigment density as well.

The change in field size produces systematic shifts in the peak optical density of photopigments due to its self-screening effect (as in CIEPO06). In addition, age, genetics, pupil entry, and retinal illuminance are reported to change photopigment optical density. Increasing age decreases the photopigment optical density in the fovea [63] but increases the photopigment optical density in the perifovea [26, 64]. Other authors found no age effect on the photopigment optical density [48, 65]. Elsner et al. [48] reported that the optical density for a 4° field size was minimally affected by aging. Differences in genetic sequences might regulate the photopigment optical density. Neitz et al. found a single amino-acid substitution was correlated with trichromacy in the subjects who have photopigments with the same peak wavelength sensitivity and differing only in optical density [66]. Pupil entry, although it is rare to observe under practical viewing conditions, affects color matches (Stiles & Crowford II effect) [51]. High retinal illuminance (approximately more than 8000 Td, pp.619 in [67]) decreases the photopigment optical density due to photopigment bleaching [47, 68, 69]. Note that it would rarely happen under practical viewing conditions as the luminance of 3500 [*cd*/*m*^2^] is required to reach such high retinal illuminance (assuming the typical pupil size of 3 mm in diameter).

Genetic polymorphism causes λ_*max*_ shifts of L- and M-cone photopigments. Some studies showed bimodal or multi-modal distributions in λ_*max*_ (or Rayleigh matches) [27, 70–72], which would be attributed to λ_*max*_ shift of L-cones caused by alanine/serine substitutions at codon 180. On the contrary, some studies showed no or weak multi-modality [50, 73, 74]. Moreover, not only the alanine/serine substitutions at codon 180, but other genetic sequences also exist that encode L- and M-photopigments with different λ_*max*_ [75] (also [76, 77]). They would disturb the bimodality caused by the pure L-cone alanine/serine substitutions at codon 180. It might be an indication that the distribution would eventually become unimodal when different genotypes are mixed among populations.

Note that these factors identified above (e.g., genetics, gender, etc.) could be additional model inputs for future work but were not incorporated into the proposed model since they are not well quantified yet.

## Color Matching Simulation Procedure

The procedure to simulate color matches for a given observer function is explained. For a given color match among the five matches [52], there were a reference spectrum (**S**_**ref**_, 401 × 1 vector), and spectra of three matching primaries with their maximum energy spectra (**S**_**match**,**max**_, 401 × 3 matrix). 401 is the number of wavelength samplings from 380 to 780 nm at 1 nm interval. A color match can be simulated for a given observer *k* (= a set of CMFs), **C**_**k**_ (3 × 401 matrix).

When a color match is achieved, Eq (13) holds.

$$\begin{array}{c} C\cdot S_{\text{ref}}=C\cdot S_{\text{match},\text{max}}\cdot R \end{array}$$ (13)

where **R** (3 × 1 vector) is a set of three scalars to modify the intensities of the three primaries. **R** is estimated by a matrix inversion as expressed in Eq (14).

$$\begin{array}{c} R={\left(C\cdot S_{\text{match},\text{max}}\right)}^{-1}C\cdot S_{\text{ref}} \end{array}$$ (14)

The matched spectrum (**S**_**matched**_, 401 × 1 vector) is reconstructed using the estimated scalars as in Eq (15).

$$\begin{array}{c} S_{\text{matched}}=S_{\text{match},\text{max}}\cdot R \end{array}$$ (15)

CIEXYZ values (**T**_**matched**_, 3 × 1 vector) are computed from the reconstructed spectrum and the CIE 1964 observer (**C**_**CIE1964Obs**_) as shown in Eq (16). Note that the CIE 1964 observer instead of the specified observer function (**C**_**k**_) was used to compute the tristimulus values in order to align the color space with experimental results [52].

$$\begin{array}{c} T_{\text{matched}}=683\cdot C_{\text{CIE}1964\text{Obs}}\cdot S_{\text{matched}} \end{array}$$ (16)

Finally, CIELAB values (**P**_**matched**_, 3 × 1 vector) corresponding to the matched CIEXYZ values are computed using the reference white XYZ values (**T**_**n**_, 3 × 1 vector) as shown in Eq (17).

$$\begin{array}{c} P_{\text{matched}}=f\left(T_{\text{matched}},T_{n}\right) \end{array}$$ (17)

**T**_**n**_ is computed in Eq (18).

$$\begin{array}{c} T_{n}=683\cdot C_{\text{CIE}1964\text{Obs}}\cdot S_{\text{refW}} \end{array}$$ (18)

where **S**_**ref** **W**_ is a spectrum of reference white for the specified color match. **S**_**ref** **W**_ was defined as follows. For color match 1, 2, and 5, the colors of the reference spectra were neutral. Thus, the relative spectral shape of the reference white was assumed to be same as the reference spectrum. For color match 3 and 4, the colors of the reference spectra were saturated cyan and saturated orange, respectively. Thus, the relative spectral shape of the reference white was assumed to be equal-energy spectrum (Illuminant E). For all the five color matches, the intensity of the relative spectrum of the reference white was adjusted such that CIELAB L* of the reference spectrum became 50 for the CIE 1964 observer. More details are found in Ch.3.1.3 of [52].

The procedure described above allows to simulate color matches in Asano’s Ph.D. work [52] for a given color match and a given observer function. Also, it allows to compare the simulated CIELAB values with those from experimental results.

## Validation of Physiological Parameter Deviations

The derived standard deviations were validated using three different color matching datasets. In all the datasets, SDs measured (or obtained) in an experiment were compared with SDs predicted by the proposed model. lms-CMFs were generated through Monte Carlo simulation using an age distribution and a field size of a target study as input. The process in the proposed model was that CIEPO06 sets the baseline CMFs using the supplied age and field size information and the eight additional physiological parameters randomly deviated the baseline CMFs. The number of CMF sets depended on each validation dataset. Then, color matching was simulated, and standard deviations were taken. This process was repeated 100 times and average SDs were taken to increase the accuracy of predicted SDs. Table 7 summarizes the measured and predicted SDs for three validation datasets. Matlab codes for simulating these three color matching datasets are available at Munsell Color Science Laboratory’s website [19].

**Table 7:** Validation results of the proposed vision model. SDs measured (obtained) by each study and SDs predicted by the model are listed. SD units for Stiles & Burch, Asano, and Rüfer et al. studies are rgb-CMFs space (normalized at three primaries’ wavelengths), CIELAB, and Rayleigh Match unit, respectively.

		SDs		SD Ratio
Validation Datasets	Subjects	Meas.	Pred.	(Pred./Meas.)
CMFs (Stiles & Burch)	49	0.0374	0.0355	0.95
Five Color Matches (Asano)	76	6.49	7.91	1.22
Rayleigh Match (Rüfer et al.)	113	2.7	3.1	1.15

The first dataset was the Stiles and Burch’s color matching data [1]. It includes color matching results of 49 color-normal observers at 35 measurement points, which essentially form 49 sets of CMFs. The data are available on the Colour & Vision Research Laboratory website [78]. There are 53 sets of CMFs on the website as four observers repeated experiments twice. These four observers’ results were averaged, and the averages were used in this study. Two observers out of 49 observers had incomplete data. Their data were interpolated and extrapolated. One observer missed data at two wavelengths that were extrapolated using average data from other observers. The other observer missed data at one wavelength that was linearly interpolated. A list of ages for 49 observers ranging from 16 to 55, and a field size of 10° were used for the model inputs. To compare the variations in the same unit, lms-CMFs were linearly transformed to rgb-CMFs. For each set of lms-CMFs, a 3 × 3 matrix was obtained such that the corresponding rgb-CMFs were normalized at three primaries’ wavelengths (444.4, 526.3, 645.2 nm). The measured and predicted SDs (in an absolute unit) were taken at 35 wavelengths for each RGB primary, then averaged.

The second dataset was five color matches by Asano. [52]. This dataset was the same color matches as used in the derivation step 2 but included a different set of 76 observers. There were no overlapping observers between the datasets used in the derivation and the validation. A list of ages for 76 observers ranging from 20 to 69, and a field size of 8.5° were used for the model inputs. Color matches were simulated and the CIELAB values were predicted for each set of the CMFs from the Monte Carlo simulation. The simulation procedure for a given set of CMFs is described in Color Matching Simulation Procedure. The measured and predicted SDs [CIELAB unit] were obtained by averaging SDs for five color matches for both a* b* values.

The third dataset was Rayleigh match employed in Oculus HMC anomaloscope by Rüfer et al. including 113 observers [79]. For the model inputs, a list of ages was estimated from Table 1 in [79]. Ages were ranging from 14.9 to 65.4. A field size was 2°. The reported peak wavelengths of red (700 nm), green (550 nm), and yellow (589 nm) primaries were used for color matching simulation. The measured and predicted SDs were compared in Rayleigh match unit.

Overall, the predictions are close to experimental data for all the validation datasets. The estimated SDs were different from experimental data between -5 to +22%. A two-sample F-test, was performed on the Stiles and Burch’s dataset and the five color matches’ dataset to test if the variances of two samples are equal. The test was performed with 95% confidence level and two-tailed. For the Stiles and Burch’s dataset, one sample was color matching responses at 35 wavelengths for the Stiles and Burch’s 49 observers, and the other sample was the simulated color matching responses at 35 wavelengths for 1000 CMFs generated by Monte Carlo simulation. The test was performed for each of the 105 variables (35 wavelengths x 3 primaries). The results showed the variances were significantly different for 72 variables and were not significantly different for 33 variables. For the five color matches’ dataset, one sample was the adjusted a* and b* values of five color matches for 76 observers, and the other sample was the simulated a* and b* values of five color matches for 1000 CMFs generated by Monte Carlo simulation. The test was performed for each of the 10 variables (2 values x 5 matches). The results showed the variances were significantly different for 9 variables and were not significantly different for 1 variable. The two-sample F-test was not performed on the Rayleigh match dataset since individual matching results were unavailable.

F-test results did not support statistical similarities of model predictions to the experimental data perfectly. This could be because there was experimental noise present (predicted SDs were noise-free), and factors other than age (e.g., ethnic origins, gender, genetics, etc.), which are not incorporated in the proposed model, might affect color vision variations. Nevertheless, the test results infer that at least for some variables, there are statistical similarities between the model predictions and experimental data.

It should be pointed out that, regarding the five color matches’ dataset, given that the average intra-observer variability of five color matches was 1.4 (computed from Table 3.5 in [52]), the difference between measured and predicted SDs (1.42 CIELAB unit) in Table 7 would be perceptually small. In other words, this mathematical observer model is very useful in a practical sense even if it is not yet capable of predicting each element of observed variability perfectly in a statistical sense.

**Fig 2:**
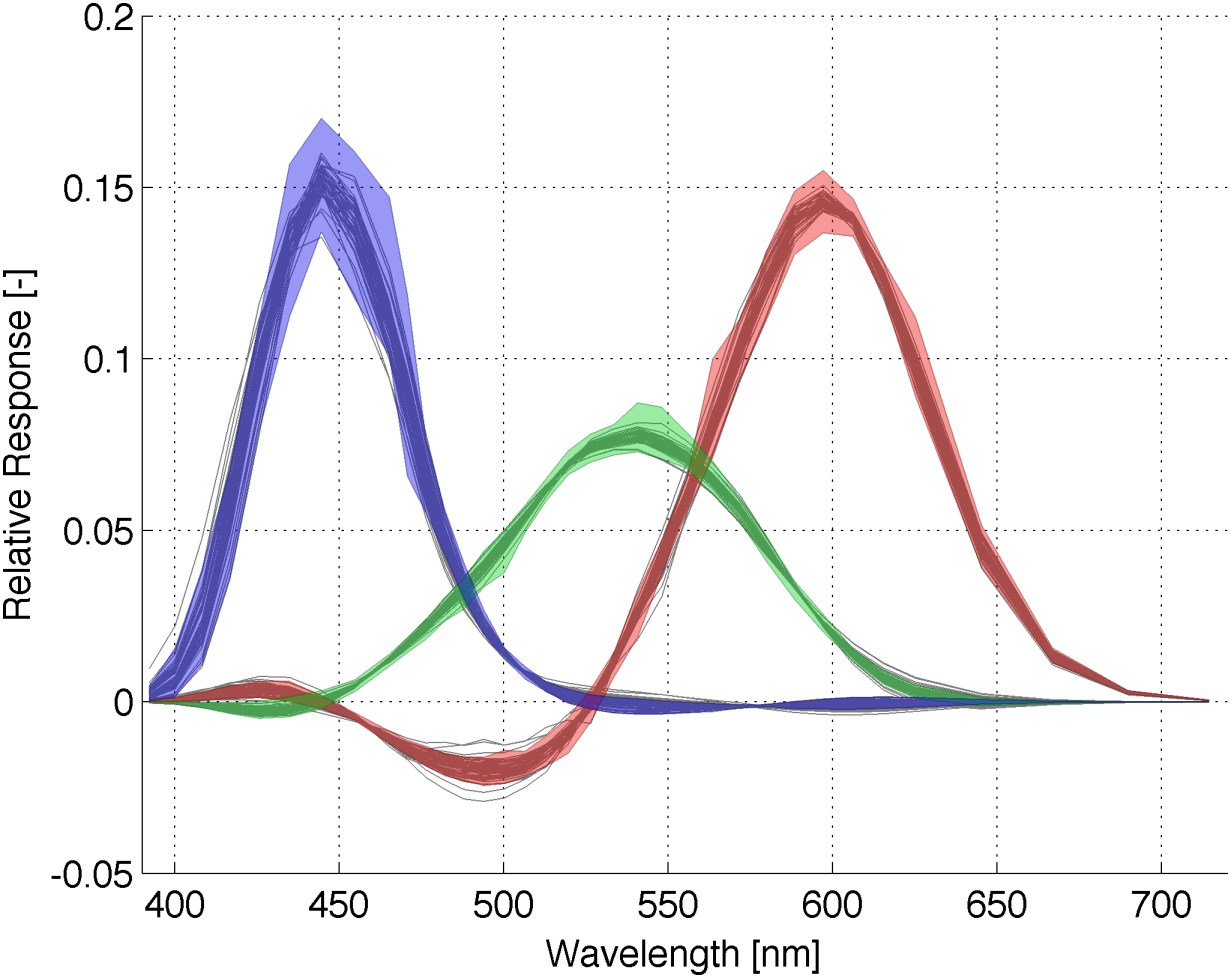
49 sets of rgb-CMFs generated by the proposed observer model (gray lines) aiming to predict the Stiles and Burch’s experiment results. The maxima and minima of 49 sets of CMFs for the Stiles and Burch’s experiment participants are superimposed as color-shaded areas. All the CMFs are normalized to equal area.

**Fig 3:**
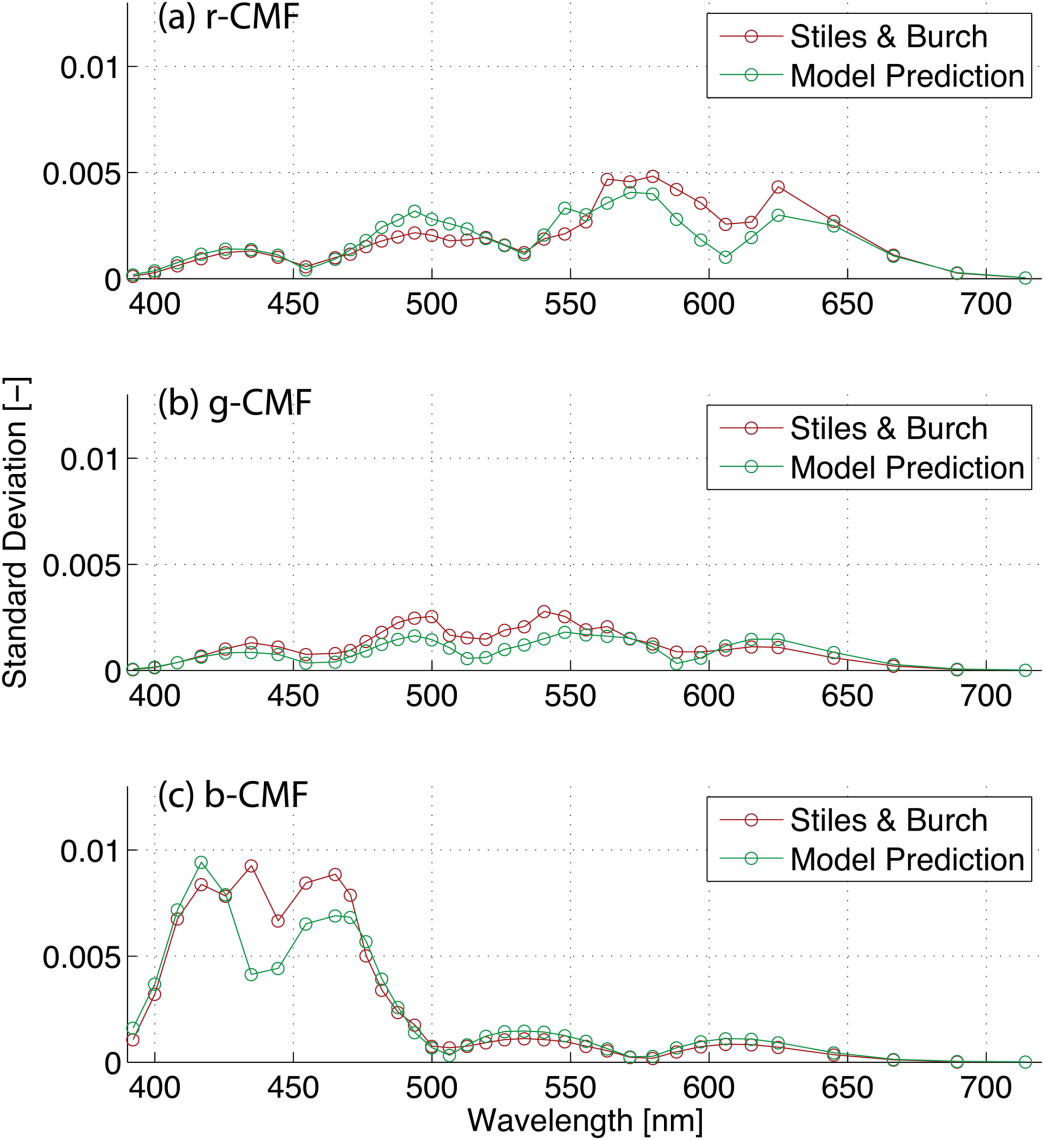
Standard deviations computed for Stiles and Burch’s 49 observers (red lines) and for 49 sets of rgb-CMFs generated by the proposed observer model (green lines). The plot (a), (b), and (c) show standard deviations in red, green, and blue CMFs, respectively. Area-normalized rgb-CMFs are used to compute standard deviations in these plots.

To visualize the measured and predicted variability, CMFs measured by Stiles and Burch and CMFs predicted by the proposed vision model were compared in Fig 2. Gray lines represent 49 sets of rgb-CMFs generated by the proposed observer model while color-shaded areas represent maxima and minima of 49 Stiles and Burch’s observers. Note that both CMFs are area-normalized to better visualize the variability. Alternatively in Fig 3, the standard deviations are plotted for Stiles and Burch’s 49 observers (red lines) and for 49 sets of rgb-CMFs generated by the proposed observer model (green lines). Overall, the variability appears practically and usefully similar between Stiles and Burch’s observers and the simulated CMFs in Figs 2 and 3.

## Conclusion

A synthetic vision model for individual colorimetric observers was proposed. It would be beneficial for simulating color matches and identifying the range of matches among color-normal populations. The model is an extension of CIEPO06 and possesses eight additional physiological parameters with corresponding standard deviations. The model can simulate CMFs that represent a population for a given age (or a given age distribution) and a field size using Monte Carlo simulation. The required steps to implement the model using Monte Carlo simulation technique are as follows. (1) Choose an age distribution and a field size as inputs, (2) Randomly pick observer age (in case an age distribution is input) and deviation values for the eight physiological parameters, and (3) Repeat to generate as many CMFs as necessary.

The variances of model input parameters were derived with two steps; standard deviations of eight physiological parameters were obtained from past studies in the first step; the obtained standard deviations were scaled down to fit color matching data in the second step. The final standard deviations are the current best estimates of inter-observer variability. The variances of model input parameters were validated using three different datasets: color matching data with 49 observers [1], five color matches with 76 observers [52], and Rayleigh matches with 113 observers [79]. The two-sample F-test partially supported the derived standard deviations. Moreover, the model prediction would be perceptually similar to actual color matching results considering noise due to intra-observer variability.

Additionally, the possible factors were identified that might affect physiological parameters and CMFs in Table 6. These factors are not incorporated into the proposed model as they have not been quantified yet.
