# Correction: Individual Colorimetric Observer Model

**DOI:** 10.1371/journal.pone.0305847

**Published:** 2024-06-14

**Authors:** The PLOS ONE Editors

## Notice of Republication

This article [1] was republished on May 3, 2024, to address an issue identified post-publication. An updated version of S1 File is provided with this notice. Please download this article again to view the correct version.

## Supporting information

S1 File Underlying Data. (ZIP)
